# Ultra-short-period perioperative pulmonary rehabilitation on short-term outcomes after surgery in smoking patients with lung cancer: a randomized clinical trial

**DOI:** 10.1097/JS9.0000000000001856

**Published:** 2024-06-21

**Authors:** Dingpei Han, Xinyi Wang, Xin Sun, Yuqin Cao, Chengqiang Li, Wei Guo, Yanxia Hu, Junbiao Hang, Jian Li, Qing Xie, Hecheng Li

**Affiliations:** a Department of Thoracic Surgery, Ruijin Hospital, Shanghai Jiao Tong University School of Medicine; b Department of Rehabilitation, Ruijin Hospital, Shanghai Jiao Tong University School of Medicine; c Clinical Research Center, Ruijin Hospital, Shanghai Jiao Tong University School of Medicine, Shanghai, People’s Republic of China

**Keywords:** lobectomy, lung cancer, pulmonary rehabilitation, randomized controlled trial

## Abstract

**Background:**

Pulmonary rehabilitation (PR) is essential for airway management after thoracic surgery. Most current PRs are composed of 2–4-week exercises, which require significant consumption of medical resources and concerns about disease progression.

**Materials and methods:**

This single-center, prospective, randomized controlled trial enrolled smoking patients with pulmonary masses or nodules suitable for lobectomy, aged 18–80, with a smoking history (≥20 pack-years). Eligible patients were randomized in a 1:1 ratio into two groups. Patients in the intervention group underwent perioperative breathing exercises based on positive pressure vibration expectoration and 3-day preoperative lower limb endurance training. Patients in the control group received routine perioperative care. The primary outcome was in-hospital incidence of postoperative pulmonary complications. Secondary outcomes included postoperative hospital stay, total hospitalization cost, postoperative drainage time, drainage volume, semiquantitative cough strength score, pain score, Borg scale‐assessed fatigue, and walking distance on postoperative days 1 and 2.

**Results:**

A total of 194 patients were included in the study, with 94 in the intervention group and 100 in the control group. Our ultrashort PR program potentially reduced pulmonary complications incidence (24.5 vs. 33.0%), but without statistical significance (*P*=0.190). No significant differences were found in other perioperative outcomes, except for postoperative semiquantitative cough strength score (3 [interquartile range, 3–3.75] vs. 3 [interquartile range, 2–3], *P*<0.001) and change in walking distance from postoperative days 1 to 2 (60 [interquartile range, 40–82.5] vs. 30 [interquartile range, 10–60], *P*=0.003).

**Conclusion:**

There were no significant differences in postoperative complications and other hospitalizations, but our ultrashort rehabilitation program improved patients’ semiquantitative cough strength score and walking distance, indicating the potential for better outcomes. This treatment is a safe and effective means of airway management for thoracic surgery in the era of enhanced recovery (ClinicalTrials.gov Identifier: NCT03010033).

## Introduction

Highlights

- Considering medical resources consumption and disease progression, whether an ultra-short-period perioperative pulmonary rehabilitation leads to improved postoperative outcomes in smoking patients with lung cancer?
- Our ultra-short-period rehabilitation program demonstrated a significant improvement in patients’ semiquantitative cough strength score and walking distance, suggesting the potential for better postoperative recovery.
- This study offers valuable insights into the efficient management of airways in thoracic surgery in the enhanced recovery after surgery era.

In 2021, the WHO released a report estimating 1.3 billion smokers globally, with tobacco use associated with 8.71 million deaths globally in 2019^1^. Tobacco-related diseases, such as chronic obstructive pulmonary disease (COPD), cerebrocardiovascular disease, and lung cancer, have become enormous burdens not only to public health but also to economic costs^2^. As nicotine products from both traditional cigarettes and heated tobacco products have the potential to increase oxidative stress and microbial adherence to the respiratory tract^3^, it was reported that preoperative smoking might increase postoperative complications in head/neck, thoracic, and abdominal surgeries^4^. In particular, smoking increases the risk of postoperative morbidity and mortality by more than 50% in patients undergoing pulmonary resection for lung cancer^5^.

Pulmonary rehabilitation (PR), first officially defined in 1981 by the American Thoracic Society, is a comprehensive intervention aimed at reducing the symptoms and functional impairment of patients with chronic respiratory disease, with treatments that include exercise training, education, nutritional and psychological counseling, and social behavior changes^6^. Airway management plays a critical role in the perioperative period of thoracic surgery, with PR serving as a vital component of airway management. Evidence has shown the clinical effects of PR in patients with COPD and other respiratory disorders such as asthma, interstitial lung disease, pulmonary hypertension, bronchiectasis, and lung cancer^7^. For patients with lung cancer, both outpatient and home-based PR programs have been shown to improve exercise capacity, reduce symptom burden, and prevent physiological impairment in patients’ quality of life^8,9^. Furthermore, postoperative PR, as a component of the enhanced recovery after surgery (ERAS) pathway, reduces postoperative pulmonary complications (PPCs) after lung resection feasibly^10^. However, it is not easy to engage patients with PR before surgery, although a meta-analysis in patients with lung cancers has demonstrated that preoperative PR efficiently reduced hospital length of stay and PPCs^11^ because it tends to be urgent for patients to ask for definitive treatment once a diagnosis of lung cancer is made. Therefore, it is important to recommend PR programs only for high-risk patients before pulmonary resection for lung cancer. Patients with over 20 pack-years of smoking history, age >70 years, BMI >30, and COPD are at especially high-risk for postoperative airway complications after thoracic surgery^12^.

In 2022, an expert consensus proposed a clinical pathway for perioperative PR in patients with lung cancer combined with COPD, based on a large quantity of clinical evidence^13^. However, little is known about the effects of perioperative PR in heavy smokers with lung cancer, and whether smoking cessation is required before lung resection is still controversial^14^. Moreover, the current several-week PR program may be too long for cancer patients, because of the potential risk of disease progression and healthcare resource consumption. Therefore, we designed a perioperative PR program that included a 3-day preoperative PR program for smokers with resectable lung cancer. A single-center, prospective randomized controlled trial (RCT) was conducted to determine whether the PR program effectively improved patients’ short-term postoperative results. To the best of our knowledge, this is the first RCT to report the impact of a perioperative PR program that included an ultrashort preoperative period for smoking patients with lung cancer.

## Methods

### Study design and participants

This single-center, prospective, randomized, controlled clinical trial compared perioperative PR with regular airway management in smokers undergoing lobectomy. This study was initiated and conducted by the Department of Thoracic Surgery, Ruijin Hospital, in accordance with the Declaration of Helsinki. This study was approved by the Ethics Committee Ruijin Hospital in November 2016 (2016-129) and registered at ClinicalTrials.gov (NCT03010033). Written informed consent was obtained from all the participants. The trial protocol appears in SDC, Supplement 1 (Supplemental Digital Content 1, http://links.lww.com/JS9/C849). This study is a primary analysis reported in line with the Consolidated Standards of Reporting Trials (CONSORT) reporting guideline^15^.

### Inclusion and exclusion criteria

Patients with pulmonary masses or nodules identified as suitable for open or minimally invasive lobectomies were included in this study. Eligible patients were aged 18–80 years with a history of smoking (≥20 pack-years). The exclusion criteria were as follows: (1) poor pulmonary function [forced expiratory volume in 1 s (FEV_1_)/forced vital capacity (FVC) <0.7, FEV_1_ <50% predicted]; (2) severe brain, heart, kidney, or liver dysfunction; (3) inability to cooperate; (4) stage IV lung cancer with distant metastasis; (5) need for emergency surgery; (6) history of preoperative chemotherapy, radiotherapy, or chemoradiotherapy for lung cancer; and (7) pathologically confirmed a benign lesion.

### Trial procedures

Preoperatively, all patients were asked to cease smoking at least 1 week before surgery while receiving routine symptomatic treatment, if necessary, and regular rehabilitation education. In addition to these measures, patients in the intervention group were trained in prescribed respiratory and lower limb endurance exercises. This was achieved using lower extremity endurance training (using a bike ergometer for 3 days, 2 times/day, 15–20 min/time or stair climbing training for 3 days, 2 times/day, 30 min/time), and inspiratory muscle training (using a threshold inspiratory muscle trainer for 3 days, 5 times/day, 2 sessions/time; every session includes 10–20 cycle respirations). All exercises were supervised by a physiotherapist.

After lobectomy using either an open or minimally invasive surgical approach, the control group received regular postoperative treatments, including antibiotics, pain control, oxygen therapy, nebulized expectorants, bronchodilators, chest pat three times per day, and earliest ambulation. In addition to the treatments listed above, the interventional group also received PR featuring the utilization of inspiratory muscle training (using a threshold inspiratory muscle trainer until hospital discharge, 3–5 times/day, 1 session/time; every session includes 10–20 cycle respirations).

### Outcomes

The primary endpoint was the in-hospital incidence of PPCs, including (1) pneumonia, (2) atelectasis, (3) empyema, (4) prolonged air leak, (5) pleural effusion, and (6) respiratory failure. Pneumonia was defined as at least four of the following criteria: Leukocytosis >15×10^9^/l, temperature >38°C, cough and expectoration, pulmonary rales, and pulmonary infiltrates on chest radiograph. A prolonged air leak was defined as a persistent air leak requiring chest tube drainage for greater than 5 days after surgery. Secondary endpoints included postoperative hospital stay, total hospitalization cost, postoperative drainage time, drainage volume, semiquantitative cough strength score (SCSS), pain score, Borg scale-assessed fatigue, and walking distance on postoperative day (POD)1 and POD2. Cough strength was measured using the SCSS graded from 0 (weak) to 5 (strong)^16^. The postoperative pain score was evaluated and recorded using the visual analog scale (VAS). Walking distance was defined as the maximum distance that a participant could walk with the assistance of a physiotherapist. During the process, the participants’ vital signs remained stable, and oxygen saturation was maintained at over 90%. The total time of walking should not exceed 10 min.

### Sample sizes

The sample size was estimated based on the hypothesis that PR can reduce pulmonary complications in smokers after lobectomy, which was the primary endpoint. According to a published study that determined the effects of positive pressure vibration expectoration after thoracic surgery^17^, the incidence of pulmonary complications was 25.7% in the control group and 10.0% in the intervention group. The required sample size of each arm (ratio=1:1) was calculated as 93 cases to detect a reduction in pulmonary complications from 25.7% to 10.0% based on a bilateral significance level (β) of 0.05, and a power of test (1-β) of 0.80. Allowing for a drop-out rate of 10%, the estimated sample size was 200 (100 patients per arm).

### Randomization and concealment

After signing an informed consent form, all eligible participants were randomized into the control group or intervention group using the method of permutated block randomization. Adequate allocation concealment before randomization further prevents selection bias. Blinding was not performed by the patients, surgeons, or physical therapists, due to the nature of the rehabilitation intervention.

### Statistical analysis

Continuous variables are presented as mean±SD, and Student’s *t*-test was used for comparison when data were normally distributed. Non-normally continuous variables were presented as median [interquartile range (IQR)] and compared between groups using the Wilcoxon rank-sum test. The *χ* ^2^ test or Fisher’s exact test was used for categorical variables. The test level between the two groups was set at α=0.05 (bilateral), and *P*<0.05 was considered to be statistically significant. All analyses were performed using R software (version 4.1.2; R Foundation for Statistical Computing).

## Results

### Baseline characteristics of patients

**Figure 1:**
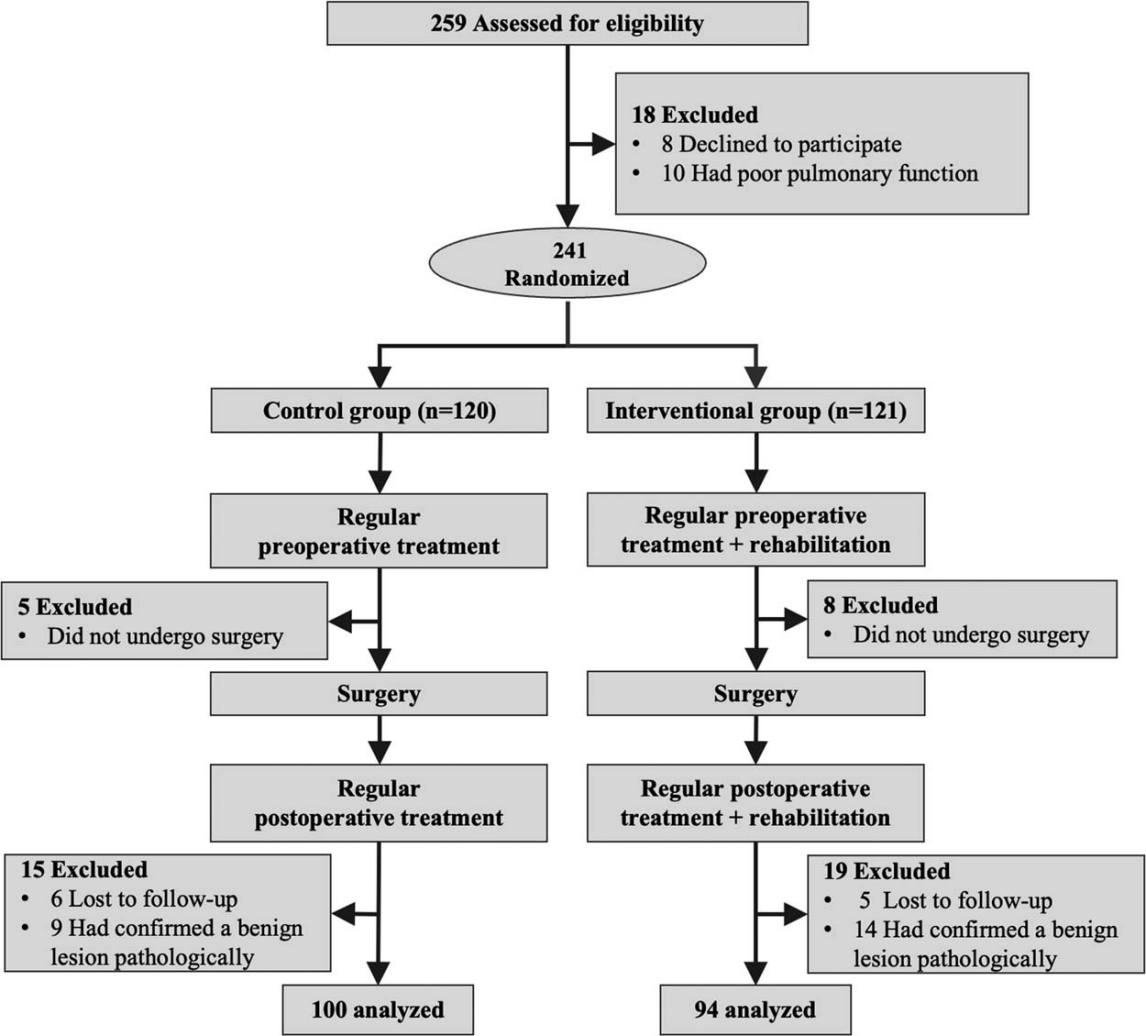
Study enrollment.

Of the 259 patients assessed for eligibility from November 2016 to April 2023, eight declined to participate in our PR program and 10 had poor pulmonary function. A total of 241 patients were enrolled in this study and randomly assigned to the PR and control groups. Thirteen patients did not undergo surgery and 11 lacked postoperative follow-up information. Furthermore, 23 patients with pathologically confirmed benign lesions were excluded. Ultimately, 94 patients in the intervention group and 100 in the control group were included in the analysis (Fig. 1).

The median (IQR) age of all enrolled patients was 64 (57–69) years and all participants were male. The median (IQR) BMI was 24.13 (22.50–25.93) kg/m^2^. A total of 166 (85.6%) patients underwent lobectomy (84 [43.3%] in the control group and 82 [42.3%] in the intervention group), and 28 (14.4%) underwent segmentectomy or wedge resection. Most patients (143 [73.7%]) underwent video-assisted thoracic surgery (VATS), 36 [18.6%] underwent robot-assisted thoracic surgery (RATS), and 15 [7.7%] underwent thoracotomy. Most enrolled patients had adenocarcinoma (141 [72.7%]). The majority of tumors in both groups were pathological stage IA (52 [55.3%] in the intervention group and 67 [67.0%] in the control group). A majority of patients (160 [82.4%]) underwent mediastinal lymph node dissection and no significant differences were observed in the type of lymph node retrieval between these two groups (84.0% vs. 81.0%, *P*=0.577). Patient demographics and baseline characteristics are summarized in Table 1 and were comparable in both groups.

**Table 1:** Patient demographic and baseline characteristics.

Characteristic	Intervention (*N*=94)	Control (*N*=100)	*P*
Sex, No. (%)			
Male	94 (100)	100 (100)	
Age [years], median (IQR)	64 (57–69)	63.5 (57–69)	0.600
≤60	40 (42.6)	35 (35.0)	0.280
>60	54 (57.4)	65 (65.0)	
BMI [kg/m^2^], median (IQR)	24.17 (22.54–25.57)	23.97 (22.39–26.10)	0.899
ECOG, No. (%)			0.286
0	63 (67.0)	74 (74.0)	
1	31 (33.0)	26 (26.0)	
ASA, No. (%)			0.299
1	0 (0)	2 (2.0)	
2	74 (78.7)	82 (82.0)	
3	20 (21.3)	16 (16.0)	
FEV1/FVC, median (IQR)	0.793 (0.748–0.859)	0.788 (0.743–0.841)	0.373
FEV1%pred, mean (±SD)	0.860 (±0.150)	0.870 (±0.161)	0.648
Surgical technology, No. (%)			0.356
Thoracotomy	8 (8.5)	7 (7.0)	
VATS	65 (69.1)	78 (78.0)	
RATS	21 (22.3)	15 (15.0)	
Surgical type, No. (%)			0.927
Lobectomy	80 (85.1)	82 (82.0)	
Segmentectomy	4 (4.3)	6 (6.0)	
Wedge resection	8 (8.5)	10 (10.0)	
Lobectomy+segmentectomy/wedge resection	2 (2.1)	2 (2.0)	
Location of surgery, No. (%)			0.606
Left	39 (41.5)	38 (38.0)	
Right	54 (57.4)	62 (62.0)	
Both	1 (1.1)	0 (0)	
Lymph node, No. (%)			0.577
Dissection	79 (84.0)	81 (81.0)	
sampling	15 (16.0)	19 (19.0)	
Pathology, No. (%)			0.810
Adenocarcinoma	67 (71.3)	74 (74.0)	
Squamous carcinoma	21 (22.3)	19 (19.0)	
Others	3 (3.2)	5 (5.0)	
Metastatic cancer	3 (3.2)	2 (2.0)	
Stage, No. (%)			0.159
0	2 (2.1)	4 (4.0)	
IA1	23 (24.5)	23 (23.0)	
IA2	21 (22.3)	32 (32.0)	
IA3	8 (8.5)	12 (12.0)	
IB	9 (9.6)	7 (7.0)	
IIA	5 (5.3)	1 (1.0)	
IIB	9 (9.6)	8 (8.0)	
IIIA	14 (14.9)	7 (7.0)	
IIIB	0 (0)	4 (4.0)	
Metastatic	3 (3.2)	2 (2.0)	
Diabetes, No. (%)			0.176
No	75 (79.8)	87 (87.0)	
Yes	19 (20.2)	13 (13.0)	
Hypertension, No. (%)			0.283
No	56 (59.6)	67 (67.0)	
Yes	38 (40.4)	33 (33.0)	
Cardiovascular disease, No. (%)			0.082
No	79 (84.0)	93 (93.0)	
Yes	15 (16.0)	7 (7.0)	
Respiratory disease, No. (%)			0.492
No	92 (97.9)	95 (95.0)	
Yes	2 (2.1)	5 (5.0)	
History of tumor, No. (%)			0.386
No	83 (88.3)	92 (92.0)	
Yes	11 (11.7)	8 (8.0)	
History of thoracic surgery, No. (%)			0.623
No	90 (95.7)	98 (98.0)	
Yes	4 (4.3)	2 (2.0)	

ASA, American Society of Anesthesiologists; ECOG, Eastern Cooperative Oncology Group; FEV1, forced expiratory volume in 1 second; FVC, forced vital capacity; IQR, interquartile range.

### Effect of PR on the incidence of PPCs

There were no deaths in either group during the 30-day study period. Table 2 compares the postoperative complication rates between the two groups. The incidence of PPCs in the intervention group (23/94, 24.5%) was lower than that in the control group (33/100, 33.0%); however, this difference was not statistically significant (*P*=0.190). The most frequent complications were pleural effusion (19.1% vs. 16.0%; *P*=0.698), prolonged air leakage (10.6% vs. 13.0%; *P*=0.611), and pneumonia (12.8% vs. 12.0%; *P*=0.871). However, these differences were not statistically significant.

**Table 2:** Thirty-day postoperative complication rates.

Characteristic	Intervention (*N*=94)	Control (*N*=100)	*P*
PPCs, No. (%)			0.190
No	71 (75.5)	67 (67.0)	
Yes	23 (24.5)	33 (33.0)	
Pneumonia, No. (%)			0.871
No	82 (87.2)	88 (88.0)	
Yes	12 (12.8)	12 (12.0)	
Atelectasis, No. (%)			0.247
No	94 (100)	97 (97.0)	
Yes	0 (0)	3 (3.0)	
Empyema, No. (%)			1.000
No	94 (100)	99 (99.0)	
Yes	0 (0)	1 (1.0)	
Prolonged air leak, No. (%)			0.611
No	84 (89.4)	87 (87.0)	
Yes	10 (10.6)	13 (13.0)	
Pleural effusion, No. (%)			0.698
No	76 (80.9)	84 (84.0)	
Yes	18 (19.1)	16 (16.0)	
Respiratory failure, No. (%)			1.000
No	94 (100)	99 (99.0)	
Yes	0 (0)	1 (1.0)	
Thirty-day readmission, No. (%)			0.696
No	85 (90.4)	93 (93.0)	
Yes	9 (9.6)	7 (7.0)	

PPCs, postoperative pulmonary complications.

### Effect of PR on perioperative outcomes

All secondary outcomes of the two groups are summarized in Table 3. No significant differences were found in the duration of chest tube placement (4 days [IQR, 3–5 days] vs. 4 days [IQR, 3–6 days], *P*=0.559), drainage of chest tube on POD1 to POD3 (*P*>0.05), VAS (3 [IQR, 2–4] vs. 3 [IQR, 2–4], *P*=0.474), Borg score (2 [IQR, 1–2] vs. 2 [IQR, 1–2], *P*=0.121), walking distance (95 m [IQR, 42.5–120 m] vs. 60 m [IQR, 30–120 m], *P*=0.472), postoperative hospital stay (5 days [IQR, 4–6 days] vs. 5 days [IQR, 4–6.25 days], *P*=0.619), or total hospitalization cost (65863.51 ¥ [IQR, 56156.97–80048.56 ¥] vs. 61860.15 ¥ [IQR, 53512.01–73675.66 ¥], *P*=0.149). SCSS was significantly higher in the intervention group than in the control group (3 [IQR, 3–3.75] vs. 3 [IQR, 2–3], *P*<0.001).

**Table 3:** Perioperative outcomes.

Characteristic	Intervention (*N*=94)	Control (*N*=100)	*P*
Chest tube duration [days], median (IQR)	4 (3–5)	4 (3–6)	0.559
Chest tube drainage [ml], median (IQR)			
POD 1	285 (155.0–397.5)	285 (137.5–425.0)	0.910
POD 2	220 (132.5–315.0)	220 (150.0–365.5)	0.669
POD 3	130 (70.0–217.5)	145 (52.5–240.0)	0.974
SCSS, median (IQR)	3 (3.00–3.75)	3 (2.00–3.00)	<0.001
VAS, median (IQR)	3 (2–4)	3 (2–4)	0.474
Borg score, median (IQR)	2 (1–2)	2 (1–2)	0.121
Walking distance [meter], median (IQR)	95 (42.5–120.0)	60 (30.0–120.0)	0.472
Postoperative hospital stay [days], median (IQR)	5 (4.00–6.00)	5 (4.00–6.25)	0.619
Hospitalization cost [¥], median (IQR)	65863.51 (56156.97–80048.56)	61860.15 (53512.01–73675.66)	0.149

IQR, interquartile range; POD, postoperative day; SCSS, semiquantitative cough strength score; VAS, visual analog scale.

We selected patients with follow-up information on POD2, and compared the relevant indicators on POD2 with those on POD1 (Table 4). The results showed that the pain score, Borg score, and walking distance were significantly improved on POD2 compared with POD1 in both groups. However, no significant differences were observed in changes in the pain score (1 [IQR, 1–2] vs. 1 [IQR, 0–2], *P*=0.917) or Borg score (0.5 [IQR, 0–1] vs. 1 [IQR, 0–1], *P*=0.110). The improvement in walking distance on POD2 compared to POD1 in the intervention group was much more significant than that in the control group (60 [IQR, 40–82.5] vs. 30 [IQR, 10–60], *P*=0.003).

**Table 4:** Comparison of rehabilitation-related indicators.

Characteristic	Intervention (*N*=36)	Control (*N*=24)	*P*
VAS (POD1), median (IQR)	3 (2–4)	3 (3–4)	0.545
Borg (POD1), median (IQR)	2 (1–3)	2 (2–3)	0.130
Walking distance (Day1) [meter], median (IQR)	60.0 (25.0–120.0)	60.0 (22.5–60.0)	0.190
VAS (POD2), median (IQR)	2 (1–3)	2 (2–3)	0.302
Borg (POD2), median (IQR)	1 (1–2)	1 (1–2)	0.428
Walking distance (Day2) [meter], median (IQR)	120.0 (80.0–172.5)	82.5 (60.0–120.0)	0.001
−△VAS, median (IQR)	1 (1–2)	1 (0–2)	0.917
−△Borg, median (IQR)	0.5 (0–1.0)	1.0 (0–1.0)	0.110
△Walking distance [meter], median (IQR)	60.0 (40.0–82.5)	30.0 (10.0–60.0)	0.003

IQR, interquartile range; POD, postoperative day; SCSS, semiquantitative cough strength score; VAS, visual analog scale.

### Subgroup analysis of patients undergoing lobectomy

We performed additional analysis only on patients who underwent lobectomy. These results are presented in Supplementary eTables 1–3 (in SDC, Supplement 2, Supplemental Digital Content 1, http://links.lww.com/JS9/C849). Similarly, the incidence of postoperative complications in the intervention group was reduced, but the reduction was not statistically significant (28.0% vs. 36.9%, *P*=0.223). Patients in the intervention group had better coughing ability than those in the control group (3 [IQR, 3–4] vs. 2.5 [IQR, 2–3], respectively, *P*<0.001).

## Discussion

Smoking is not only an important risk factor for the development of lung cancer, but also for postoperative complications. Mason *et al*.^18^ retrospectively analyzed 7990 primary resections for lung cancer in adults. The results showed that the mortality and pulmonary complication rates of smokers were 1.5 and 6.2%, respectively, whereas those of nonsmokers were only 0.39 and 2.5%, respectively. Additionally, smoking status was found to be an independent risk factor for postoperative pain^19^. An analysis of expert consensus suggests that it is right to recommend smoking cessation, but wrong to require it in consideration of disease progression and alienating patients^20^. Long-term preoperative exercise is the main reason for the limited application of PR before lung cancer surgery. In this study, we made full use of the preoperative time to perform our ultrashort PR program, which could benefit patients without increasing hospitalization costs.

The results of our study showed that our ultrashort PR program potentially reduced the incidence of pulmonary complications (23/94, 24.5% vs. 33/100, 33.0%), but the result did not show a significant difference (*P*=0.190). Similar results were also shown in the subgroup analysis of patients undergoing lobectomy. There are several possible explanations for this observation. First, we excluded patients with poor pulmonary function, who were more likely to benefit from PR. Second, the development of minimally invasive surgery has substantially reduced postoperative complications^21^, with minimally invasive surgery (including VATS and RATS) accounting for 92.2% of the cases in this study. Third, the prevalence of ERAS was commonly applied to patients in our center, including those in the control group in this study. The routine ERAS pathway has been shown to significantly decrease postoperative complications, length of hospital stay, and hospitalization costs^22^. The patients in both groups underwent perioperative management following routine ERAS protocols. These factors may have resulted in an insignificant difference in the PPC rate between the two groups.

Cough weakness is also associated with extubation failure^23^. Our results showed that postoperative SCSS was higher in the intervention group than in the control group, which indicated that our rehabilitation program could improve patients’ ability to cough, indicating the potential for better outcomes. Respiratory muscle training is essential for PR to improve diaphragm and intercostal muscle strength. The diaphragm is responsible for 60–80% of all respiratory work. Many factors can lead to diaphragmatic weakness, including COPD and obesity^24^. The reduced lung function in smokers may result from reduced maximal voluntary activation and force generation in the diaphragm^25^. The threshold inspiratory muscle train used in our study is a commonly used device that combines positive expiratory pressure (PEP) and high-frequency oscillation therapy for respiratory muscle training. The repeated inspiratory effort was effective because of an extended inspiration mechanism that could induce an equal pressure point to migrate proximally, preventing early airway closure at the peripheral airways^26^. The two-phase gas flow process during the breathing maneuver may alter the rheological properties of mucus, improving its clearance^27^. Additionally, resistance training has been shown to increase intrathoracic pressure, which enhances bronchial drainage and expectoration^28^. This may account for the improved coughing or expectoration abilities of the patients in the PR group.

Our study found that postoperative improvement in walking distance was more significant in patients receiving perioperative PR. Short-term preoperative high-intensity interval training (HIIT) significantly increases patients’ 6 min walking distance^29^. A few HIIT sessions appear to be sufficient to accelerate transcriptional mitochondrial biogenesis, resulting in aerobic phenotypic alterations marked by an increase in the expression and activity of proteins involved in fat oxidation, the tricarboxylic acid cycle, and the electron transport chain in the mitochondria^30^. While not as intense as HIIT, our perioperative rehabilitation program indicated that 3-day exercises may help enhance postoperative walking ability.

In our PR program, patients were trained under the guidance of professional physiotherapists to avoid insufficient rehabilitation and the potential dangers caused by home-based rehabilitation. Most previous studies that included aerobic exercise comprised 2–4 weeks of intervention^31,32^, while our program was carried out 3 days before surgery, which did not impose an additional burden on patients. No patients dropped out of the program. However, in other studies, some patients did not complete the entire rehabilitation training or withdrew due to intolerance or lack of benefit^33^. This may reflect the fact that patients are more willing to accept ultra-short-period PR programs with the avoidance of extra time and additional burdens^12^.

This study has several limitations. Most patients included in our study underwent lobectomy; thus, for safety considerations in surgical patients, surgeons pre-excluded high-risk populations (such as those with COPD). In addition, no women were recruited in this study, which may limit the generalizability of our findings; however, this is in part due to the low smoking prevalence among women^34^. Similar sex-related biases have been observed in other clinical studies focusing on smoking patients^35,36^. Furthermore, blinding was not performed in our research, which might introduce potential bias in the assessment of outcomes. Considering that SCSS was based on subjective evaluations by healthcare professionals, and walking distance can also be influenced by various factors such as patient motivation and pain tolerance, we recognize that potential biases may have influenced these measurements.

## Conclusions

In conclusion, our study confirmed that the ultra-short-period rehabilitation program was safe and effective and could improve patients’ SCSS and walking ability postoperatively, indicating the potential for better outcomes. This study provides an efficient method of airway management in thoracic surgery in the ERAS era. Further investigations with larger sample sizes are still needed to more firmly establish its effectiveness.

## Ethical approval

Ruijin Hospital Ethics Committee, Shanghai Jiao Tong University School of Medicine Ethics Committee Reference Number: 2016-129.

## Consent

Informed consent of patients was obtained, and no personal information of patients appeared in the data.

## Source of funding

This study was supported by National Natural Science Foundation of China (82072557), National Key Research and Development Program of China (2021YFC2500900), Shanghai Municipal Education Commission – Gaofeng Clinical Medicine Grant (20172005, the 2nd round of disbursement), a program of Shanghai Academic Research Leader from Science and Technology Commission of Shanghai Municipality (20XD1402300), Novel Interdisciplinary Research Project from Shanghai Municipal Health Commission (2022JC023), and Interdisciplinary Program of Shanghai Jiao Tong University (YG2023ZD04).

The study sponsors had no such involvement.

## Author contribution

Mr Li had full access to all of the data in the study and takes responsibility for the integrity of the data and the accuracy of the data analysis; D.H. and H.L.: concept and design; D.H., X.W., X.S., Y.C., C.L., W.G., J.L., and H.L.: acquisition, analysis, or interpretation of data; D.H., X.W., and X.S.: drafting of the manuscript; D.H., X.W., and J.L.: statistical analysis; H.L.: obtained funding; D.H., X.W., X.S., Y.C., C.L., W.G., Y.H., J.H., Q.X., and H.L.: administrative, technical, or material support; D.H. and H.L.: supervision. All authors contributed in critical revision of the manuscript for important intellectual content.

## Conflicts of interest disclosure

The authors declare no conflict of interest.

## Research registration unique identifying number (UIN)

ClinicalTrials.gov Identifier: NCT03010033.

## Guarantor

Hecheng Li.

## Data availability statement

Interested investigators will be required to submit a formal letter of intent outlining research aims, rationale, and approach to the corresponding author. Furthermore, documentation of local IRB approval, including a description of type of review, should be submitted with the data request.

## Provenance and peer review

Not commissioned, externally peer-reviewed.

## Supplementary Material
