# Supplementary material for: Ultra-short-period perioperative pulmonary rehabilitation on short-term outcomes after surgery in smoking patients with lung cancer: a randomized clinical trial
Source: Int J Surg. 2024 Jun 21;111(1):581–8. doi: 10.1097/JS9.0000000000001856 (PMC11745579; doi:10.1097/JS9.0000000000001856)
Supplement: Supplementary file 1 [file js9-111-0581-s001.pdf]

Ultra-short-period Perioperative Pulmonary Rehabilitation on Short-term Outcomes after Surgery in Smoking Patients with Lung Cancer

A Randomized Clinical Trial

Protocol

Corresponding Author:

Hecheng Li, MD, PhD, FACS

Department of Thoracic Surgery

Ruijin Hospital, Shanghai Jiao Tong University School of Medicine

197 Ruijin 2nd Road

Shanghai 200025, China

E-mail: lihecheng2000@hotmail.com

Tel: +86-021-64370045

Abstract

Objective

Smoking has become one of the biggest public health problems around the world. It is reported that smoker patients will suffer more postoperative complications than non-smoker patients after major surgeries. There is evidence that pulmonary rehabilitation can improve the short-term outcomes after thoracic surgery. However, the effects and long-term results of perioperative pulmonary rehabilitation on smoker patients have not been widely studied.

Design

This single-institutional prospective randomized controlled parallel-group clinical trial compares perioperative pulmonary rehabilitation with regular airway management in smoker patients undergoing lobectomy. Incidence of postoperative pulmonary complications is regarded as the primary endpoint. Perioperative changes in postoperative hospital stay, total hospitalization cost, postoperative drainage time and etc. are also evaluated.

Conclusion

The purpose of this study is to determine whether pulmonary rehabilitation is effective for smoker patients undergoing lobectomy and to provide an optimization for enhanced recovery after surgery.

Key Words

Pulmonary rehabilitation • Smokers • Lung neoplasms • Lobectomy

Background

The World Health Organization (WHO) launched a report in 2019 that estimates 1.1 billion adult smokers globally. In China, the age-standardized prevalence of tobacco use in 2017 is estimated at 48% among males and 1.8% among females, indicating a smoker population over 300 million. Tobacco has become one of the world's biggest public health problems, killing more than 8 million people every year as a result of both direct tobacco use and exposure to second-hand smoke[1].

Smoking has proved to be an independent risk factor of postoperative complications[2]. It is reported that smoking can significantly increase the incidence of pulmonary, cardiac and neurologic complications after major surgeries and has a negative effect on the incision healing and pain control[2-4].

Recently, a series of strategies for airway management including pulmonary rehabilitation have been proposed by clinicians. The evidence-based medicine has demonstrated that positive pressure vibration expectoration can increase the breathing capacity and reduce complications after thoracic surgery[5] while lower limb resistance training can improve muscle strength and endurance[6]. However, the small sample size of participants and short period of follow-up have limited the quality of existing evidence and its impact on clinical practice.

Therefore, this randomized controlled trial has been designed to determine whether perioperative pulmonary rehabilitation, including the use of positive pressure vibration expectoration and preoperative lower limb endurance training, is effective for improving short-term as well as long-term outcomes in smoker patients undergoing lobectomy who had a significant decrease in lung function.

Methods

Objectives

This study aims to evaluate the effectiveness of perioperative pulmonary rehabilitation on enhanced recovery after surgery (ERAS) in smoker patients undergoing lobectomy.

Study design

This is a single-institutional prospective randomized controlled parallel-group clinical trial comparing perioperative pulmonary rehabilitation with regular airway management in smoker patients with lung cancer. The study is initiated and conducted by the Department of Thoracic Surgery, Shanghai Jiao Tong University School of Medicine Affiliated Ruijin Hospital in accordance with the Declaration of Helsinki. It has been approved by the Ethics Committee of Ruijin Hospital and registered on the site of ClinicalTrials.gov (NCT03010033). Written informed consent was obtained from each participating patient.

Sample size calculation

The sample size was estimated with the hypothesis that pulmonary rehabilitation can reduce pulmonary complications in smoker patients after lobectomy, which serves as the primary endpoint. According to a recently published study determining the effects of positive pressure vibration expectoration after thoracic surgery[5], the incidence of pulmonary complications in the control group was 25.7% while it was 10.0% in the interventional group. The required sample size of each arm (ratio=1:1) was calculated as 93 cases to detect the reduction in the pulmonary complications from 25.7% to 10.0% based on a bilateral significance level (α) of 0.05 and a power of test ($1-\beta$) of 0.80. Considering an estimated drop rate of 10%, the minimum sample size of this study is 200 patients, 100 in each group.

Study population

All smoker patients up to 80 years old that are suspected of having lung cancer and who undergo lobectomy in the Department of Thoracic Surgery, Shanghai Jiao Tong University School of Medicine Affiliated Ruijin Hospital are assessed for eligibility. The inclusion criteria and exclusion criteria are as follows:

Inclusion criteria

- Age ≤ 80 years.

- Smoking history (≥ 20 pack years).
- Suspected lung neoplasm.
- Tumors resectable by open or minimally-invasive lobectomy.
- Written informed consent.

Exclusion criteria

- $FEV_1/FVC < 0.7$ and FEV_1 <50% predicted.
- Severe brain, heart, kidney, or liver dysfunction.
- Inability to cooperate.
- Stage IV lung cancer with distant metastasis.
- Need for emergency surgery.
- Preoperative chemotherapy, radiotherapy or chemoradiotherapy for lung cancer.
- Pathologically confirmed a benign lesion.

Randomization

After signing the informed consent, every eligible participant is randomized into control group or interventional group based on permutated block randomization. Blinding is not applied to patients, surgeons, or physical therapists.

Preoperative treatment

Patients in the control group are asked to cease smoking at least one week before surgery while receiving nebulized expectorants and bronchodilators, regular rehabilitation education, and antibiotics if necessary preoperatively.

In addition to these measures, patients in the pulmonary rehabilitation group are trained with prescribed respiratory and lower limb endurance exercises. The vibratory positive expiratory pressure (PEP) therapy system Acapella (PORTEX, Smiths Medical, USA) is used for 3 days before surgery (10-20 cycles per session, 2 sessions per time, 5 times per day) along with 15-20 minutes of bike ergometry (twice per day) or 30 minutes of stair climbing (twice per day) for 3 days. All the exercises are supervised by a physiotherapist and the Modified Borg Dyspnea Scale (MBS) measures are maintained in a range of 5 to 7.

Postoperative treatment

After lobectomy by either open or minimally-invasive surgical approach, the control group receives regular postoperative treatments that include antibiotics, pain control, oxygen therapy, nebulized expectorants and bronchodilators, chest pat 3 times per day and earliest ambulation.

Besides the treatments above, the interventional group also receives pulmonary rehabilitation featuring the utilization of a vibratory PEP therapy system Acapella, which combines PEP therapy and airway vibration to mobilize pulmonary secretions. The equipment is used 3-5 times per day (10-20 cycles per session, 1 session per time) until discharge. Patients are recommended to continue the respiratory muscle training after discharge.

Outcome measurements and follow-up

The primary endpoint is the in-hospital incidence of postoperative pulmonary complications. The secondary outcome measurements include postoperative hospital stay, total hospitalization cost, postoperative drainage time, drainage volume, semiquantitative cough strength score (SCSS), pain score, Borg scale-assessed fatigue, and walking distance on postoperative day (POD)1 and POD2

Statistical analysis

The number of postoperative pulmonary complications will be recorded and the chi-squared test will be used to compare the incidences between the control and interventional groups. The Student's *t*-test will be applied to evaluate the differences between the two groups for continuous variables that follow a normal distribution while the nonparametric test will be used for non-normally continuous variables. Differences are considered statistically significant if $P < 0.05$.

Discussion

According to the WHO report on the tobacco epidemic, the global smoker population has reached 1.1 billion in 2019. Every year, more than 7 million smokers are killed by direct tobacco use while around 1.2 million non-smokers are killed by exposure to second-hand smoke[1]. Meanwhile lung cancer, serving as the leading cause of cancer-related death worldwide, can be related to a high smoking prevalence[7].

Not only a promoter of oncogenesis but also a risk factor for postoperative complication has proved smoking. Turan et al.[3] conducted a propensity-matched study involving 82,304 current smokers and 82,304 never-smoker controls and concluded that 30-day mortality was significantly increased in smoker patients with an odds ratio (OR) of 1.38 (95% CI, 1.11–1.72) after non-cardiac surgeries. Current smokers also showed greater likelihood of developing serious postoperative complications, ranging from pneumonia (OR, 2.09; 95% CI, 1.80–2.43) to stroke (OR, 1.73; 95% CI, 1.18–2.53). Other large population studies have demonstrated a negative effect of smoking on pulmonary, cardiac and neurologic complications, incision healing and pain control as well[2,4]. Therefore, airway management plays an important role in the postoperative recovery especially for smokers who account for around 30% of patients receiving surgery every year.

As one of the essential parts of airway management, pulmonary rehabilitation aims to reduce disability in patients with lung disease and to improve their quality of life while diminishing the health care burden[8]. Previously most studies had focused on the efficacity and feasibility of pulmonary rehabilitation on chronic obstructive pulmonary disease (COPD)[9-13]. In the recent years, a series of innovations have been applied to the perioperative airway management of thoracic surgery with the concept of ERAS[14,15]. The introduction of positive pressure vibration expectoration machine has relieved the burden of medical care and improved the performance of sputum excretion. A prospective study performed by Huang et al. demonstrated that short term application of Acapella Duet after thoracic surgery reduced the incidence of pulmonary complications by 15.7% (control group, 25.7%; interventional group, 10.0%; $P<0.05$) while increasing the 24-hour sputum volume (control group, 15.53±3.11 V/mL; interventional group, 24.29±3.51 V/mL; $P<0.05$), shortening the hospital stay (control

group, 10.61±2.25 days; interventional group, 8.61±1.58 days; $P<0.05$) and duration of drainage (control group, 5.96±2.04 days; interventional group, 4.36±1.57 days; $P<0.05$)[5]. Meanwhile, lower limb resistance training has showed improvements in muscle strength as well as exercise endurance in several clinical trials[6,16,17]. However, the quality of existing evidence and its impact on clinical practice is limited due to the relatively small sample size and short period of follow-up.

Aiming to determine the effects of perioperative pulmonary rehabilitation on short-term outcomes after lobectomy, this prospective randomized controlled parallel-group clinical trial has been designed. With the hypothesis that pulmonary rehabilitation can reduce postoperative pulmonary complications and improve the recovery of pulmonary function, the perioperative pulmonary rehabilitation is regarded as a promising intervention to optimize the ERAS program in thoracic surgery, especially for the large number of smoker patients.

Conclusion

This is a single-institutional prospective randomized controlled trial comparing perioperative pulmonary rehabilitation with regular airway management in postoperative outcomes for smoker patients with lung cancer. It is hypothesized that pulmonary rehabilitation may have a positive effect on complication rates and lung function recovery after lobectomy.

Trial status

This study was approved by the Ethics Committee of Shanghai Jiao Tong University School of Medicine Affiliated Ruijin Hospital in November 2016 and was registered on the site of ClinicalTrials.gov in January 2017. The recruitment of participants was finished in April 2023.

References

1. World Health Organization G. WHO Report on the Global Tobacco Epidemic, 2019. *Licence: CC BY-NC-SA 30 IGO.* 2019.
2. Schmid M, Sood A, Campbell L, et al. Impact of smoking on perioperative outcomes after major surgery. *Am J Surg*. 2015;210(2):221-229.e6
3. Turan A, Mascha EJ, Roberman D, Roberman D, Turner PL, You J, Kurz A, Sessler DI, Saager L. Smoking and perioperative outcomes. *Anesthesiology*. 2011;114(4):837-846.
4. Duan G, Guo S, Zhang Y, Ying Y, Huang P, Zhang L, Zhang X. Effects of Epidemiological Factors and Pressure Pain Measurements in Predicting Postoperative Pain: A Prospective Survey of 1,002 Chinese Patients. *Pain Physician*. 2017; 20(6):E903-E914.
5. Huang Q, Jia Z, Song L. Effect of positive pressure vibration expectoration machine Acapella Duet on the sputum excretion and lung function of patients after thoracic surgery. *China Medical Herald.* 2016;13(14):158-161+169.
6. Chen Y, Niu M, Zhang X, Qian H, Xie A, Wang X. Effects of home-based lower limb resistance training on muscle strength and functional status in stable Chronic obstructive pulmonary disease patients. *J Clin Nurs.* 2018;27(5-6):e1022-e1037.
7. Islami F, Torre LA, Jemal A. Global trends of lung cancer mortality and smoking prevalence. *Transl Lung Cancer Res*. 2015;4(4):327-38.
8. British Thoracic Society Standards of Care Subcommittee on Pulmonary Rehabilitation. Pulmonary rehabilitation. *Thorax.* 2001;56(11):827-834.
9. Barbarito N, Vaghi A, De Mattia E. Prevalence of airflow obstruction according GOLD, ATS and ERS criteria in symptomatic ever-smokers referring to a pulmonary rehabilitation department. *Monaldi Arch Chest Dis.* 2011;75(3):157-161.
10. Keating A, Lee A, Holland AE. What prevents people with chronic obstructive pulmonary disease from attending pulmonary rehabilitation? A systematic review. *Chron Respir Dis.* 2011;8(2):89-99.
11. Moore L, Hogg L, White P. Acceptability and feasibility of pulmonary rehabilitation for COPD: a community qualitative study. *Prim Care Respir J.* 2012;21(4):419-424.
12. Hayton C, Clark A, Olive S, Browne P, Galey P, Knights E, Staunton L, Jones A, Coombes E, Wilson AM. Barriers to pulmonary rehabilitation: characteristics that predict patient attendance and adherence. *Respir Med.* 2013;107(3):401-407.
13. Sahin H, Naz I. Why are COPD patients unable to complete the outpatient pulmonary rehabilitation program?. *Chron Respir Dis.* 2018;15(4):411-418.
14. Louie AV, Palma DA, Dahele M, Rodrigues GB, Senan S. Management of early-stage non-small cell lung cancer using stereotactic ablative radiotherapy: controversies, insights, and changing horizons. *Radiother Oncol.* 2015;114(2):138-147.
15. Wang JY, Hong X, Chen GH, Li QC, Liu ZM. Mucosolvan serves to optimize perioperative airway management for NSCLC patients in fast track surgery: a randomized placebo controlled study. *Eur Rev Med Pharmacol Sci.* 2015;19(15):2875-2881.
16. Sillen MJ, Franssen FM, Delbressine JM, Vaes AW, Wouters EF, Spruit MA. Efficacy of lower-limb muscle training modalities in severely dyspnoeic individuals with COPD and quadriceps muscle weakness: results from the DICES trial. *Thorax.* 2014;69(6):525-531.
17. De Brandt J, Spruit MA, Hansen D, et al. Changes in lower limb muscle function and muscle

mass following exercise-based interventions in patients with chronic obstructive pulmonary disease: A review of the English-language literature. *Chron Respir Dis.* 2018;15(2):182-219.

eTable 1. Baseline characteristics of patients receiving lobectomy

Characteristic	Intervention (N=82)	Control (N=84)	*P* value
**Gender, No. (%)**			
Male	82 (100)	84 (100)	
**Age [years], median (IQR)**	64 (57.25-69.00)	64 (57.75-69.25)	0.62
≤60	34 (41.5)	27 (32.1)	0.278
>60	48 (58.5)	57 (67.9)	
**BMI [kg/m^2], median (IQR)**	24.125 (22.535-25.380)	24.080 (22.085-26.153)	0.79
**ECOG, No. (%)**			0.785
0	55 (67.1)	59 (70.2)	
1	27 (32.9)	25 (29.8)	
**ASA, No. (%)**			0.34
1	0 (0)	2 (2.4)	
2	63 (76.8)	67 (79.8)	
3	19 (23.2)	15 (17.9)	
**FEV1/FVC, median (IQR)**	0.787 (0.742-0.859)	0.788 (0.743-0.844)	0.529
**FEV1%pred, mean (±SD)**	0.856 (±0.157)	0.860 (±0.159)	0.872
**Surgical technology, No. (%)**			0.52
Thoracotomy	8 (9.8)	7 (8.3)	
VATS	54 (65.9)	62 (73.8)	
RATS	20 (24.4)	15 (17.9)	
**Location of surgery, No. (%)**			0.634
Left	33 (40.2)	31 (36.9)	
Right	48 (58.5)	53 (63.1)	
Both	1 (1.2)	0 (0)	
**Lymph node, No. (%)**			0.675
Dissection	73 (89.0)	73 (86.9)	
sampling	9 (11.0)	11 (13.1)	
**Pathology, No. (%)**			0.633
Adenocarcinoma	55 (67.1)	59 (70.2)	
Squamous carcinoma	21 (25.6)	19 (22.6)	
Others	3 (3.7)	5 (6.0)	
Metastatic cancer	3 (3.7)	1 (1.2)	
**Stage, No. (%)**			0.133
0	2 (2.4)	2 (2.4)	
IA1	14 (17.1)	13 (15.5)	
IA2	18 (22.0)	29 (34.5)	
IA3	8 (9.8)	12 (14.3)	
IB	9 (11.0)	7 (8.3)	
IIA	5 (6.1)	1 (1.2)	
IIB	9 (11.0)	8 (9.5)	
IIIA	14 (17.1)	7 (8.3)	
IIIB	0 (0)	4 (4.8)	

Metastatic	3 (3.7)	1 (1.2)	
**Diabete, No. (%)**			0.363
No	66 (80.5)	73 (86.9)	
Yes	16 (19.5)	11 (13.1)	
**Hypertension, No. (%)**			0.445
No	50 (61.0)	57 (67.9)	
Yes	32 (39.0)	27 (32.1)	
**Cardiovascular disease, No. (%)**			0.144
No	68 (82.9)	77 (91.7)	
Yes	14 (17.1)	7 (8.3)	
**Respiratory disease, No. (%)**			0.459
No	80 (97.6)	79 (94.0)	
Yes	2 (2.4)	5 (6.0)	
**History of tumor, No. (%)**			0.401
No	72 (87.8)	78 (92.9)	
Yes	10 (12.2)	6 (7.1)	
**History of thoracic surgery, No. (%)**			0.656
No	78 (95.1)	82 (97.6)	
Yes	4 (4.9)	2 (2.4)	

Abbreviations: ASA, American Society of Anesthesiologists; BMI, body mass index; ECOG, Eastern Cooperative Oncology Group; FEV1, forced expiratory volume in 1 second; FVC, forced vital capacity; IQR, interquartile range; SD, standard deviation.

eTable 2. Thirty-day postoperative complication rates of patients receiving lobectomy

**Characteristic**	**Intervention (N=82)**	**Control (N=84)**	***P* value**
**PPCs, No. (%)**			0.223
No	59 (72.0)	53 (63.1)	
Yes	23 (28.0)	31 (36.9)	
**Pneumonia, No. (%)**			0.949
No	70 (85.4)	72 (85.7)	
Yes	12 (14.6)	12 (14.3)	
**Atelectasis, No. (%)**			0.252
No	82 (100)	81 (96.4)	
Yes	0 (0)	3 (3.6)	
**Empyema, No. (%)**			1.000
No	82 (100)	83 (98.8)	
Yes	0 (0)	1 (1.2)	
**Prolonged air leak, No. (%)**			0.541
No	72 (87.8)	71 (84.5)	
Yes	10 (12.2)	13 (15.5)	
**Pleural effusion, No. (%)**			0.388
No	64 (78.0)	70 (83.3)	
Yes	18 (22.0)	14 (16.7)	
**Respiratory failure, No. (%)**			1.000
No	82 (100)	83 (98.8)	
Yes	0 (0)	1 (1.2)	
**Thirty-day readmission, No. (%)**			0.564
No	73 (89.0)	77 (91.7)	
Yes	9 (11.0)	7 (8.3)	

Abbreviations: PPCs, postoperative pulmonary complications.

eTable 3. Perioperative outcomes of patients receiving lobectomy

**Characteristic**	**Intervention (N=82)**	**Control (N=84)**	***P* value**
**Chest tube duration [days], median (IQR)**	4 (3-5)	4 (3-7)	0.467
**Chest tube drainage [ml], median (IQR)**			
POD 1	315 (200.0-427.5)	310 (189.0-442.5)	0.952
POD 2	220 (152.5-327.5)	230 (150.0-370.0)	0.812
POD 3	145 (82.5-247.5)	170 (70.0-252.5)	0.965
**SCSS, median (IQR)**	3 (3-4)	2.5 (2.0-3.0)	<0.001
**VAS, median (IQR)**	2.5 (2.0-4.0)	3.0 (2.0-4.0)	0.253
**Borg score, median (IQR)**	2 (1-2)	2 (1-2)	0.076
**Walking distance [meter], median (IQR)**	95.0 (42.5-120.0)	60.0 (30.0-120.0)	0.242
**Postoperative hospital stay [days], median (IQR)**	5 (4-7)	5 (4-8)	0.596
**Hospitalization cost [￥], median (IQR)**	67192.05 (58659.94-80622.51)	63383.24 (54641.61-77038.52)	0.107

Abbreviations: IQR, interquartile range; POD, postoperative day; SCSS, semiquantitative cough strength score; VAS, visual analog scale.
